# An Environmentally Friendly Practice Used in Olive Cultivation Capable of Increasing Commercial Interest in Waste Products from Oil Processing

**DOI:** 10.3390/antiox9060466

**Published:** 2020-06-01

**Authors:** Irene Dini, Giulia Graziani, Francalisa Luisa Fedele, Andrea Sicari, Francesco Vinale, Luigi Castaldo, Alberto Ritieni

**Affiliations:** 1 Department of Pharmacy, University of Naples Federico II, Via Domenico Montesano 49, 80141 Napoli, Italy; luigi.castaldo2@unina.it (L.C.); ritialb@unina.it (A.R.); 2 Linfa scarl, Via Zona Industriale Porto San Salvo, 89900 Vibo Valentia, Italy; ricerca@laboratoriolinfa.it (F.L.F.); andrea@laboratoriolinfa.it (A.S.); 3 Department of Veterinary Medicine and Animal Productions, University of Naples Federico II, Via Federico Delpino 1, 80137 Napoli, Italy; francesco.vinale@ipsp.cnr.it; 4 Institute for Sustainable Plant Protection, National Research Council, Via Università 133, 80055 Portici (NA), Italy; 5 Department of Clinical Medicine and Surgery, University of Naples Federico II, Via S. Pansini 5, 80141 Napoli, Italy; 6 Unesco Chair for Health Education and Sustainable Development, 80131 Napoli, Italy

**Keywords:** *Trichoderma* spp., EVOO, olive pomace, olive vegetation water, *Olea europea* var Leccino, HRMS-Orbitrap, phenolic identification, antioxidant activity

## Abstract

In the Rural Development Plan (2014–2020), the European Commission encouraged the conversion and supported the maintenance of organic farming. Organic olive oil (bioEVOO) production involves the use of environmentally sustainable fertilizers and the recycling of olive pomace (Pom) and olive vegetation waters (VW) to reduce the environmental impact of these wastes. An ecofriendly way to recycle olive wastes is to reuse them to extract bioactive compounds. In this study, the total phenolic compounds content, their profile and dosage, the antioxidant action in oil, pomace, and vegetation water was evaluated when the *Trichoderma harzianum* M10 was used as a biostimulant in agriculture. Two spectrophotometric tests (2,2-diphenyl-1-picrylhydrazyl (DPPH) and 2,2′-azinobis (3-ethylbenzothiazoline-6-sulfonic) acid (ABTS)) evaluated the antioxidant potential of samples, a spectrophotometric method estimated total phenolic content, and an Ultra-High-Performance Liquid Chromatography (UHPLC)–Orbitrap method evaluated the phenolics profile. Our results showed that the biostimulation improved the antioxidant potential and the total concentration of phenolics in the bioEVOO and bio-pomace (bioPom) samples and mainly enhanced, among all classes of phenolic compounds, the production of the flavonoids and the secoiridoids. Moreover, they demonstrated the *Trichoderma* action in the mevalonate pathway to produce phenols for the first time. The decisive action of the *Thricoderma* on the production of phenolic compounds increases the economic value of the waste materials as a source of bioactive compounds useful for the pharmaceutical, cosmetic, and food industries.

## 1. Introduction

The chemical composition of olives depends on the type of cultivar, pedoclimatic factors, and agricultural practices [1]. In general, olives contain oil (18–28%), the olive pulp (30–35%), and the vegetation water (40–50%) [2]. Olive oil extraction in olive mills by mechanical procedures determines some residues, solid and liquid, with a high organic weight detrimental to the environment. The nature of these wastes depends on the extraction system used to extract the olive oil. The most commonly employed are centrifugation systems (two-phase and three-phase) that produce extra virgin olive oil, a solid cake (olive pomace), and olive vegetation water [3]. The olive pomace is made in large amounts, leading to significant management problems. It contains fragments of skin, stone, pulp, olive kernel, a complex mixture of organic (lipids, carbohydrates, hemicellulose, cellulose, lignin, protein), inorganic compounds, (potassium, magnesium, calcium), and phenolic compounds [4]. Generally, it is further extracted (∼2% of pomace by weight) by solid–liquid extraction (hexane), solvent recycling, and distillation. However, the extraction process produces potent pollutants to obtain the residual oil [5]. Ecofriendly ways to recycle pomace is to use it to produce biogas [4] as animal feed, insecticide, herbicide, and compost after thermal concentration [6]. Moreover, three-phase centrifugation and pressure systems produce significant liquid waste, which is called olive vegetation water (VW) or mill wastewater. VW properties vary significantly with the type of climatic conditions, the process, and region of origin. VW causes the disposal of environmental problems due to its phenolic composition and high organic load with limited biodegradability [7]. Olive oil, the olive pomace, and the olive vegetation water contain secoiridoids, phenolic alcohols, phenolic acids, and flavonoids [8,9,10]. They are phenolic compounds with high antioxidant properties. The antioxidants are compounds able to stop or prevent the oxidation of the substrate [11]. Antioxidants are needed to prevent the formation of the reactive oxygen and nitrogen species, which cause damage to DNA, proteins, lipids, and other biomolecules [12]. Phenolics are amphiphilic compounds. In the extracts, type and dosage vary according to the higher degree of lipophilia rather than affinity with water, the matrix, and technology used to extract them. Many techniques are used alone or in a combined form to extract the phenolic compounds from olive waste products. The extraction, membrane separation, centrifugation, and chromatographic methods are usually used for this purpose. The recent patent applications obtained lower energy consumption and higher extraction efficiency with non-conventional methods such as microwaves, ultrasounds, electrotechnologies (high voltages electrical and discharges pulsed electric fields), mechanical technologies (pressurized liquid extraction), and employing supercritical fluids as an alternative of organic solvents in extraction techniques, and using reverse osmosis and tangential ultrafiltration systems in place of conventional filtration methods in membrane methods [13]. Olive oil phenols have some functional, nutraceutical, and sensory properties closely related to their chemical structure [14,15]. A higher intake of phenolic compounds reduces the risk of cardiovascular diseases [16], determines hypoglycemia, hypocholesterolemia, and hypotension, prevents angiogenesis, inflammation [17,18], and cancer [19]. Unfortunately, the phenolic compounds in olive oil waste reduce the microbial growth, rendering the organic load resistant to degradation [20,21,22]. Therefore, an attractive way to valorize olive oil waste is the possibility of recovering phenol compounds from the pomace and the vegetation water in consideration of the growing interest of the pharmaceutical, nutraceutical, cosmetical, and food industry towards sources of phenolic compounds, and to make further processing residues more readily biodegradable. This study determines the possible impact on the antioxidant activity, phenolic content and profile in the olive waste products (pomace and vegetation water) when ecofriendly biostimulation of the olive trees with *Trichoderma* M10 is used as an agronomic strategy to use them as a resource of bioactive molecules for cosmetic, food, and pharmaceutical industries. *Trichoderma* is a saprophytic living fungus that stimulates the growth of the plants, adsorbs soil pollutants such as heavy metals, improves nutrient availability, interacts with processes involved in plant responses to stress, enhances the production of phenolics and induces systemic resistance [23,24,25,26]. Previous works studied *Trichoderma*’s ability to speed up the composting process of the olive pomace and the parameters that influence the composting process [27]. To date, the effects on the concentration and type of phenols that characterize the pomace and the vegetation water, obtained from the olive oil processing when *Trichoderma* fungi are used in olive tree agriculture, are not known.

## 2. Materials and Methods

### 2.1. Plant Material

Bioformulates was tested on *Olea europaea* var. Leccino. The trees (20-year-old) situated in the South-Western Calabria (Rombiolo, Vibo Valentia, Italy) were selected and marked.

Plant material was offered d by Dr. Andrea Sicari (Linfa Scarl, Vibo Valentia, Italy). Only plants in excellent phytosanitary and nutritional status were used for experimental purposes. Six treatments were applied every month, starting from February until July. One control sample (water treatment), and 10^6^ ufc/mL of the living microbes and were applied as spray application on the leaves (10 L per row of which 5 L was sprayed and 5 L was drenching), and around the root system at 10 cm deep. Two times was replicated the field test.

### 2.2. Fungal Material

The strains *Trichoderma harzianum* (M10) (LGC Standards S.r.l. Sesto San Giovanni, Italy) were grown on potato dextrose agar medium (HiMedia, Laboratories Mumbai, Mumbai, India) and covered with sterilized mineral oil (Sigma Aldrich, St. Louis, MO, USA).

### 2.3. Oil Production

The oil samples were produced in a local three-phase mill. They were conserved in brown bottles without headspace and conserved at a constant temperature (10 ± 2 °C) until analysis.

### 2.4. Chemicals

Hydroxytyrosol was bought from Indofine (Hillsborough, NJ, USA), secologanoside was from ChemFaces Biochemical Co., Ltd. (Wuhan, China), all the other chemicals and standards were purchased from Sigma Aldrich (St. Louis, MO, USA) unless specified differently.

### 2.5. Analytical Methods

#### 2.5.1. The Phenolics Extraction

The phenolic extraction method proposed by Vasquez Roncero [28] with some modification was carried out. An amount of 25 g of oil was extracted with hexane (25 mL). The organic fraction was treated with MeOH:H_2_O/3:2 (*v*/*v*) (15 mL, three times). The extracts (three) were combined and extracted with 25 mL hexane. The hexane was dried at 40 °C in a rotary evaporator (Buchi, Switzerland); the residue was treated with 1 mL of MeOH, filtered through nylon filer (0.2 mm), frozen and stored (−18 °C) until analysis.

#### 2.5.2. Q Exactive Orbitrap LC-MS/MS Method

Ultra-High-Performance Liquid Chromatography (UHPLC, Thermo Fisher Scientific, Waltham, MA, USA) was used to the dosage of the phenolics. The chromatographic instrument was provided with an autosampler device, a Dionex degassing system (Thermo Scientific™ Ultimate 3000, Waltham, MA, USA), a quaternary UHPLC pump (1250 bar), and a column (Accucore aQ 2.6 µm 100 × 2.1 mm Thermo Scientific, Waltham, MA USA, USA) in a thermostat column compartment (T = 30 °C). The mobile phase consisted of two phases: Phase A: acetic acid (0.1%), and phase B: 100% acetonitrile. The following gradient was used for experimental purposes: 5% phase B from 0 to 5 min, 40% phase B from 6 to 25 min, 100% phase B from 25.1 to 27 min, 5% phase B from 27.1 to 35 min, 0% phase B from 35.1 to 45 min. A flow rate of 0.4 mL/min operated.

A Thermo Fisher Scientific Orbitrap LC-MS/MS (Q Exactive, Waltham, MA, USA) was employed to characterize phenolic compounds. The spectrometer was provided with a HESI II (Thermo Fisher Scientific, Waltham, MA, USA). The ion source setting parameters were: spray voltage −3.0 kV, auxiliary gas (N_2_ > 95%), sheath gas (N_2_ > 95%), auxiliary gas heater temperature 305 °C, capillary temperature 200 °C, radiofrequency that captures and focuses the ions into a tight beam S-lens RF level 50. The MS detection was performed in full scan and targeted selected ion monitoring. Full scan acquisition parameters were scan rate 2 s^−1^; scan range 100–1500 *m/z*; mass resolving power 35,000 full width at half maximum (at *m*/*z* 200); automatic gain control target 1 × 10^5^ ions; maximum injection time of 200 ms. The SIM (selected ion monitoring acquisition) parameters were: 35,000 full widths and half maximum (at *m*/*z* 200) (resolution power); 15 s (time window); 1.2 *m*/*z* (quadrupole isolation window).

#### 2.5.3. Method Validation of the Phenolics Dosage

The construction of a calibration curve was achieved using three different concentrations of each calibration standard.

The linearity of the method was obtained from the regression coefficient of the calibration curve.

Limits of detection (LODs) = 3 × $\frac{\mathrm{standard}\;\mathrm{deviation}}{angular\;coefficient}\;$

Limits of quantification (LOQs) = 10 × $\frac{\mathrm{standard}\;\mathrm{deviation}}{angular\;coefficient}$)

Intraday repeatability was performed by injecting each phenolic standard, three times, at seven different concentrations.

#### 2.5.4. Total Polyphenol Content

Total phenol content was obtained by the Folin–Ciocalteu colorimetric method described previously by Gao et al. 2000 [29]. Extracts (0.1 mL) were added to H_2_O (2 mL) and Folin–Ciocalteu reagent (0.2 mL) and were incubated at room temperature (3 min). Sequentially, 1 mL of the sodium carbonate (20%) was added, and the mixture was left (1 h) at room temperature. The total polyphenols were determined in a spectrophotometer (Lambda 25, PerkinElmer, Waltham, MA, USA) (λ = 765 nm). The results were expressed as mg gallic acid equivalents (GAE)/kg of sample. All determinations were performed in triplicate (*n* = 3).

#### 2.5.5. The Antioxidant Activity Evaluation

##### DPPH Method

The radical-scavenging capacity was performed utilizing the 2.2-diphenyl-1-picrylhydrazyl (DPPH) method proposed by Brand-Williams et al. (1995) [30]. The phenolic extract (20 μL) was dissolved in 3 mL of DPPH solution (6 × 10^−5^ mol/L), and the spectrophotometric lecture was performed every 5 min at λ = 517 nm until the steady-state (spectrophotometer Lambda 25, PerkinElmer, Waltham, MA, USA).

##### ABTS Method

2,2′-azinobis (3-ethylbenzothiazoline-6-sulfonic) acid (ABTS) procedure proposed by Re et al. was used (1999) [31]. The stock solution of reagent was obtained mixed a solution A (9.6 mg ABTS in 2.5 mL water) and 44 mL of a solution B (37.5 mg K_2_S_2_O_8_ in 1 mL H_2_O). The stock solution was conserved for 8 h in the dark at 4 °C. The work solution was performed by diluting the stock solution [1:88 (*v*/*v*)]. The dilution of the work solution was adjusted depending on the measured absorbance at λ = 734 nm, until a value between 0.7 and 0.8. The sample (100 μL) and the work solution (1 mL) were mixed, and the absorbance (λ = 734) was measured (Lambda 25, PerkinElmer, Italy) after 2 min and 30 s. Three different concentrations of 6-hydroxy-2,5,7,8-tetramethylchroman-2-carboxylic acid (Trolox) solution were used to perform the calibration curve. Results were expressed as mmol Trolox equivalent (TE) kg^−1^ FW. Triplicate experiments were done for each sample.

### 2.6. Statistical Analysis

“Statistica” software version 7.0 (StatSoft, Inc. Tulsa, OK, USA) was used to perform statistical analyses.

## 3. Results

A UHPLC-MS/MS method was employed to delineate the phenolic profile in the Extra virgin olive oil (EVOO) and olive pomace. The dosage method was validated according to AOAC instructions (AOAC 2012) [32]. Table 1 showed the parameters used to validate it.

### 3.1. The Phenolics Characterization

Seventeen phenolics, including two flavonoids, two phenolic alcohols, seven secoiridoids, and six phenolic acids, were identified and quantified. Table 2 reports the parameters used to identify phenolics in samples.

### 3.2. The Phenolics Dosage

Table 3, Table 4 and Table 5 report the dosage of each phenolic compound found in the samples. The *Trichoderma* biostimulation improved the apigenin concentration in the EVOO, and the olive pomace (Pom), but decreased it together with luteolin in VW. The Pom and the VW samples did not contain lignans (Table 3).

The phenolic acid response to the biostimulation was like that seen for the other phenols; there was an increase in the concentration of each compound, but the increase was different from compound to compound (Table 4).

Our results confirmed the *Trichoderma*’s ability to increase the concentration of secoiridoids and their degradation products in EVOO, and established a similar activity in Pom, but not in VW (Table 5 and Table 6).

### 3.3. Total Phenolic Concentration and Antioxidant Activity

The biostimulation had a positive effect on the antioxidant activity measured with both methods (ABTS and DPPH). The ABTS method evaluated the antioxidant activity of the Pom and VW more than the DPPH method, and the opposite occurred in the EVOO samples.

## 4. Discussion

**Figure 1:**
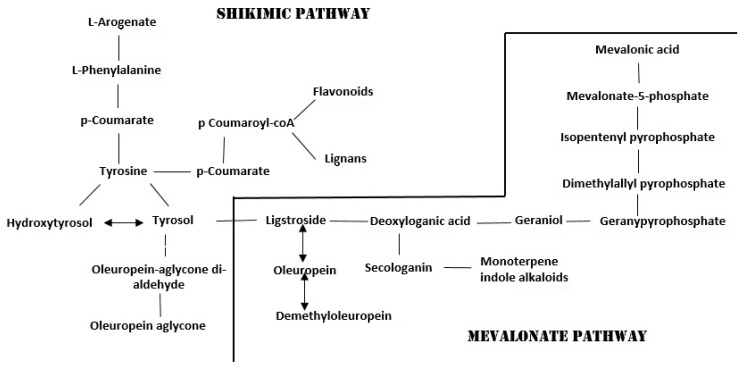
Secoiridoids biosynthesis [32].

**Figure 2:**
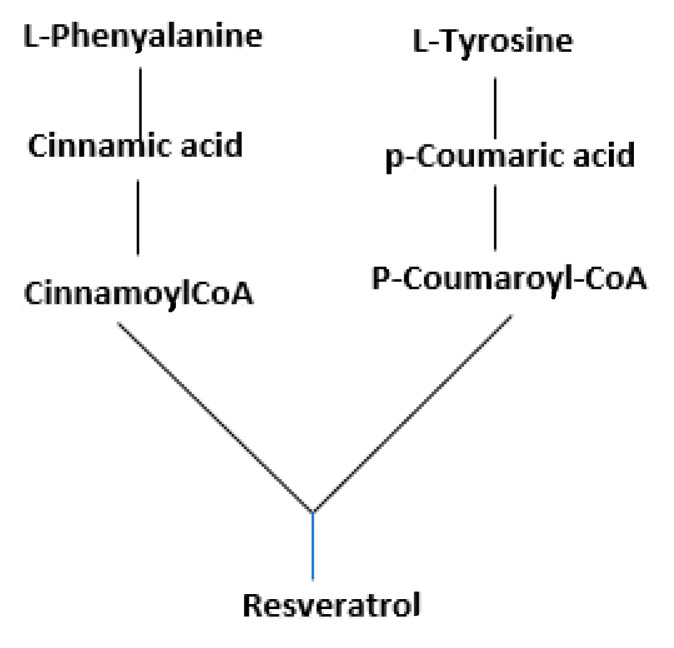
Secoiridoids biosynthesis [33].

Liquid and solid olive processing waste contain high amounts of organic materials that are not easily degradable. When these wastes are put into the environment, they create odor nuisance, an oily shine, enhance the oxygen demand, and are toxic to plant life. Therefore, the direct release of olive processing waste is forbidden, and some actions must be required before discarding into the environment. Some studies showed that olive processing waste might also be considered as an economic resource. Some practices are proposed to recycle and reuse them; using them as starting material to extract beneficial products for human health such as antioxidants is interesting. Olive pomace and olive vegetation water are sources of phenols. The industry requires phenolic compounds to produce functional foods, supplements, food additives, and the formulation of cosmetics and drugs [5,33,34]. *Trichoderma* species promote the production of phytochemicals, including phenolic compounds [23,26], whose production varies according to the strain used [35]. Previous studies have discussed that the *Trichoderma* can enhance phenols in EVOO and olive leaves [1,35]. In this work, we tested the ability of the *Trichoderma* to increase the concentration of phenolic compounds in the olive pomace and the olive vegetation water. Moreover, we determined the concentration of each compound, considering that the interest in phenolic compounds from industry depends on their chemical structure to which biological action is linked. Phenolic profile and dosage were investigated by an HPLC–Orbitrap method validated in terms of linearity, precision, and sensitivity, as recommended by the AOAC (2012) guidelines [36]. The linearity of the method was confirmed by the coefficient of regression (r ≅ 1) of the calibration curve. The sensitivity was verified by the inclusion of the concentration detected in the LODs and the LOQs range. The repeatability was confirmed by Relative Standard Deviation (RSD) values <6%. Phenolics identification was performed by comparing their mass spectra with those obtained by the standards analyses. The identification of the two hydroxybenzoic acid isomers was obtained through comparing the retention time and mass spectra with standards. The ligstroside identification, as it was not commercially available, was confirmed by comparing the chromatographic evidence and the spectroscopic data with those reported in the literature [37]. Biostimulation improved the total phenolic content in Pom and EVOO samples in accordance to our previous results [1]. On the other hand, biostimulation decreased the total phenolic concentration in VW. The main phenols found in the Pom and VW were the same as those in the EVOO, but their total concentrations, expressed as mmol Trolox/kg, were higher in the olive vegetation water control, followed by bioPom, Pom, bioVW, EVOO and bioEVOO. Among these, secoiridoids and their degradation products were the most concentrated compounds in the bioEVOO, the EVOO, and the VW samples. In contrast, the flavonoids were the most representative compounds in the bioPom, the Pom samples, and the bioVW. The lignans were found only in the bioEVOO and the EVOO. The resveratrol was in the bioVW and the VW samples. Therefore, the most variable phenolics were secologanoside, resveratrol, and lignans. Concerning secoiridoids fraction, secoiridoids biosynthesis in the plant occurs through two biosynthetic pathways: the shikimic pathway and the mevalonate pathway (Figure 1). Biostimulation with *Thricoderma* M10 enhanced the production of the oleuropein and ligstroside. It preserved them from the degradation during the malaxation process, as shown by their higher concentration and the lower concentrations of their degradation products (oleuropein-aglycone di-aldehyde, ligstroside-aglycone mono-aldehyde, tyrosol, and hydroxytyrosol) in the bioEVOO vs. the EVOO. Moreover, the higher concentration of ligstroside and secologanoside in the bioEVOO respect of the EVOO sample indicated the biostimulation’s ability to enhance the secoiridoid biosynthesis mainly through the mevalonate pathway (Figure 2). Finally, the negative variation of the percentage content of oleuropein in the pomace and strongly positive increase of its precursor, the secologanoside, in the pomace were further confirmation (Table 6).

Moreover, biocontrol agriculture enhanced the production of resveratrol, as shown by higher concentrations of the resveratrol and lower concentrations of its precursors (the cinnamic acid, and the *p*-coumaric acid) in the bioVW vs. VW sample (Figure 2). This datum is noteworthy since resveratrol has some nutraceutical properties, such as anti-inflammatory and anti-oxidative effects, and disturbs the start and progression of many illnesses such as some cancer types, and neurological and cardiovascular disorders through several mechanisms. In vitro and in vivo evidence confirmed the resveratrol’s ability as a therapeutic agent [38].

**Figure 3:**
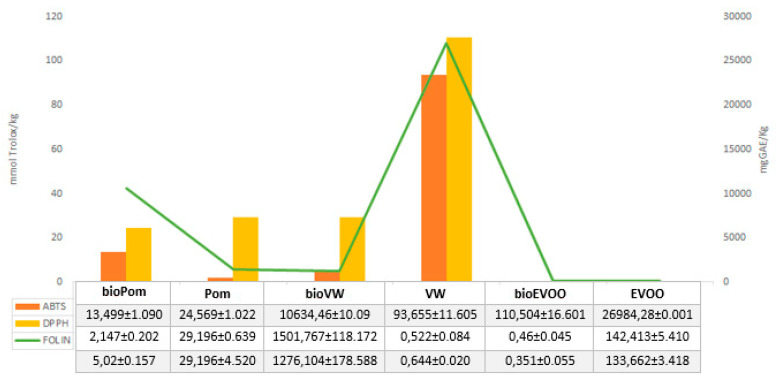
Antioxidant activities (mmol Trolox/kg) and total phenols content (mg GAE/kg)**.**

The lignans were absent in the Pom and the VW samples, as they have high solubility in fats (logP 3.1) [39]. The improvement of total phenolics concentrations, determined using the *Trichoderma* in culture, is followed by an increase in the antioxidant activity in these samples. The values of the antioxidant activity, measured with the DPPH test, are overestimated in samples where the flavonoid concentration is high (Figure 3 and Table 3) [40]. It is clear that the use of biostimulants is useful to increase the concentration of phenolic compounds with antioxidant activity, not only in the EVOO, but also in the Pom, transforming the latter from an environmental problem into a source of bioactive molecules of nutraceutical, food, pharmaceutics, and cosmetic interest. Moreover, the *Thricoderma* improves the nutraceutical value of the bioEVOO, and decreases the losses of phenolic compounds in vegetation waters, favoring the transformation of phenolic alcohols into secoiridoids, lignans, and flavonoids which have higher properties for human health.

## 5. Conclusions

For the first time, this study delineates the effects of the *Trichoderma* used in olive tree cultivation on the antioxidant activity and the phenol production in olive pomace and olive vegetation water. Our results confirmed the *Trichoderma*’s ability to improve the concentration of phenolics and antioxidant activity in EVOO, establishing the same ability in the olive pomace. Finally, they demonstrate that biostimulation principally determines, among the phenolic compounds, the biosynthesis of flavonoids and secoiridoids, two classes of phenolic compounds with well-known health properties, making olive pomace and olive vegetation water commercially appealing as a source of botanicals convenient for the food, cosmetic, and pharmaceutical industries. Finally, the *Trichoderma* action in the mevalonate pathway, producing phenols, was highlighted for the first time.

## Figures and Tables

**Table 1:** Validation parameters of the Ultra-High-Performance Liquid Chromatography (UHPLC)–MS/MS method of analysis.

Phenolic Compounds	Linearity (mg/L)	*R* ^2^	LOD (mg/L)	LOQ (mg/L)	Intraday RSD % (*n* = 3), 50 mg/L
**Phenolic Acids**					
Vanillic acid	1–50	0.887	0.200	0.600	1.1
Cinnamic acid	1–50	0.991	0.200	0.600	0.9
Ferulic acid	1–50	0.912	0.100	0.300	1.7
*p*-Coumaric acid	1–50	1.000	0.100	0.300	1.8
4-Hydroxybenzoic acid	1–50	0.998	0.207	0.622	0.9
3-Hydroxybenzoic acid	1–50	0.995	0.205	0.622	1.1
**Flavonoids and Lignans**					
Luteolin	0.5–50	0.991	0.066	0.200	1.4
Apigenin	0.5–50	0.899	0.066	0.800	2.1
*trans* Resveratrol	0.5–5.0	0.898	0.090	0.200	1.8
(+)Pinoresinol	1–50	0.999	0.02	0.060	0.5
(+)1-Acetoxypinoresinol	1–50	0.899	0.233	0.700	1.5
**Secoiridoids and Derivatives**					
Oleuropein	1–50	0.991	0.166	0.500	5.0
Ligstroside	1–50	0.991	0.166	0.500	4.0
Secologanoside	1–50	0.967	0.333	1.000	2.1
Elenaic acid	1–50	0.991	0.333	1.000	0.7
Oleacein Oleuropein-aglycone monoaldehyde	1–50	0.998	1.000	3.000	2.1
Ligstroside-aglycone dialdehyde	1–50	0.899	0.416	1.250	3.0
Tyrosol	1–50	0.991	0.133	0.040	1.6
Hydroxytyrosol	1–50	0.992	0.666	2.000	3.0

**Table 2:** Parameters used to characterize the phenolic compounds.

Phenolic Compounds	RT (min)	Formula	Theoretical *m*/*z* of Deprotonated Molecular Ions [M − H]^−^	Experimental *m*/*z* of Deprotonated Molecular Ions [M − H]^−^	Calculated Errors ∆ppm	Fragments	Collision Energy (eV)
**Phenolic Acids**							
Vanillic acid	4.30	C_8_H_8_O_4_	167.03498	167.03522	1.44	152.01143	20
Cinnamic acid	11.54	C_9_H_8_O_2_	147.04515	147.04536	1.43	103.04501	20
Ferulic acid	11.81	C_10_H_10_O_4_	193.05063	193.05084	1.09	178.02685	20
*p*-Coumaric acid	9.71	C_9_H_10_O_5_	163.04007	163.04028	1.29	119.05023	20
4-Hydroxybenzoic acid	2.57	C_7_H_6_O_3_	137.02442	137.02456	1.02	93.03431	12
3-Hydroxybenzoic acid	2.88	C_7_H_6_O_3_	137.02442	137.02458	1.17	93.03431	12
**Flavonoids and Lignans**							
Luteolin	19.07	C_15_H_10_O_6_	285.04046	285.04106	2.10	133.02940	30
Apigenin	19.12	C_15_H_10_O_5_	269.04555	269.04597	1.56	225.05592	35
*trans* Resveratrol	16.65	C_14_H_12_O_3_	227.07137	227.07147	0.44	185.06082	30
(+) Pinoresinol	17.00	C_20_H_22_O_6_	357.13436	357.13487	1.43	151.03961	40
(+) 1-Acetoxypinoresinol	19.10	C_22_H_24_O_8_	415.13984	415.14007	0.55	415.13821	40
**Secoiridoids and Derivatives**							
Oleuropein	16.69	C_25_H_32_O_13_	539.17701	539.17767	1.22	377.12393	20
Ligstroside	18.25	C_25_H_32_O_12_	523.18210	523.18279	1.32	361.12914	12
Secologanoside	19.49	C_16_H_21_O_11_	389.1092	389.109258	0.59	345.1195	12
Elenaic acid	13.14	C_11_H_14_O_6_	241.07176	241.07212	1.49	209.04573	10
Oleacein	16.14	C_17_H_20_O_6_	319.11871	319.11898	0.85	301.1082	15
Oleuropein-aglycone mono-aldehyde	21.25	C_19_H_22_O_8_	377.12419	377.12442	0.61	345.09790	12
Ligstroside-aglycone dialdehyde	18.59	C_17_H_20_O_5_	303.12380	303.12441	2.01	301.1082	12
Tyrosol	2.75	C_8_H_10_O_2_	137.06080	137.06096	1.17	119.05022	12
Hydroxytyrosol	1.60	C_8_H_10_O_3_	153.05572	153.05580	0.52	123.04561	12

**Table 3:** Flavonoids and lignans concentrations (mg/kg).

Compounds	Flavonoids			Lignans	
	Luteolin	Apigenin	*trans* Resveratrol	Pinoresinol	Acetoxipinoresinol
bioEVOO	7.317 ± 0.054	0.251 ± 0.005		0.203 ± 0.013	9.829 ± 0.035
EVOO	3.178 ± 0.046	0.228 ± 0.001		0.095 ± 0.007	4.344 ± 0.097
bioPom	110.371 ± 8.478	9.623 ± 1.011			
Pom	71.1713 ± 2.6	8.025 ± 0.27			
bioVWr	0.051 ± 0.002	0.008 ± 0.00	0.492 ± 0.0		
VW	1216.521 ± 57.985	154.388 ± 9.771	0.296 ± 0.001		

bioEVOO (Organic olive oil); EVOO (Extra virgin olive oil); Pom (pomace); bioPom (Organic pomace); VW (vegetation waters); bioVW (Organic vegetation waters).

**Table 4:** Phenolic acids concentrations (mg/kg).

Compounds	4-Hydroxybenzoic Acid	3-Hydroxybenzoic Acid	Vanillic Acid	*p*-Coumaric Acid	Cinnamic Acid	Ferulic Acid
bioEVOO	0.883 ± 0.007	0.796 ± 0.004	7.05 ± 0.059	3.274 ± 0.024	0.482 ± 0.009	0.131 ± 0.001
EVOO	0.605 ± 0.007	0.27 ± 0.003	2.663 ± 0.012	1.422 ± 0.021	0.438 ± 0.002	0.064 ± 0.000
bioPom	0.657 ± 0.016	5.033 ± 0.516	22.104 ± 3.615	21.391 ± 1.769	0.206 ± 0.02	1.486 ± 0.153
Pom	0.331 ± 0.009	2.407 ± 0.100	10.121 ± 0.11	6.085 ± 0.447	0.301 ± 0.031	0.649 ± 0.044
bioVWr	3.587 ± 0.272	0.174 ± 0.021	0.331 ± 0.035	0.238 ± 0.001	0.469 ± 0.023	0.211 ± 0.023
VW	42.146 ± 1.14	27.259 ± 1.184	116.588 ± 19.641	163.859 ± 10.169	7.092 ± 0.659	14.132 ± 0.427

**Table 5:** Secoiridoid compounds and their degradation product concentrations (mg/kg).

Compounds	Ligstroside	Oleuropein	Secologanoside	Elenaic Acid	Oleuropein-Aglycone di-Aldehyde	Ligstroside-Aglycone mono-Aldehyde	Tyrosol	Hydroxytyrosol
bioEVOO	0.009 ± 25.038	0.152 ± 2.6	0.307 ± 9.109	3.46 ± 6.552	344.531 ± 5.578	117.220 ± 2.866	105.91 ± 1.698	0.595 ± 17.946
EVOO	0.003 ± 2.205	0.099 ± 1.9	0.297 ± 1.635	7.58 ± 22.919	587.819 ± 5.041	157.254 ± 1.435	45.064 ± 6.736	0.152 ± 0.424
bioPom	0.763 ± 0.120	0.810 ± 0.09	27.724 ± 1.467	34.992 ± 0.802	17.492 ± 0.762	0.9144 ± 0.059	0.9144 ± 0.0059	8.481 ± 0.163
Pom	0.3093 ± 0.02	1.733 ± 0.005	0.519 ± 0.021	8.673 ± 0.275	2.247 ± 0.110	0.201 ± 0.0	0.201 ± 0.0	1.029 ± 0.001
bioVWr	0.0668 ± 0.003	0.484 ± 0.068	12.136 ± 0.473	0.815 ± 0.016	3.342 ± 0.111	0.0	0.014 ± 0.005	0.0
VW	3.007 ± 0.369	19.683 ± 1.245	892.645 ± 38.554	164.577 ± 8.116	9.721 ± 3.544	0.0	10.331 ± 0.989	22.678 ± 0.678

**Table 6:** Variation % of the concentration of each phenolic under biostimulation.

Compounds	Luteolin	Apigenin	Resveratrol	Pinoresinol	Acetoxypinoresinol	4-Hydroxybenzoic Acid	3-Hydroxybenzoic Acid	Vanillic Acid	*p*-Coumaric Acid	Cinnamic Acid	Ferulic Acid
bioEVOO	+130%	+10%	±	+114%	+126%	+46%	195%	165%	+130%	+10%	+105%
bioPom	−85%	+20%	+52%		±	+99%	+109%	+118%	+252%	−32%	+129%
bioVW	−100%	−100%	+66%			−92%	−99%	−100%	−100%	−93%	−99%
**Compounds**	**Ligstroside**	**Oleuropein**	**Secologanoside**	**Elenaic Acid**	**Oleuropein-Aglycone di-Aldehyde**	**Ligstroside-Aglycone mono-Aldehyde**	**Tyrosol**	**Hydroxytyrosol**			
bioEVOO	+219%%	+68%	+3%	−51%	−41%	−26%	−77%	+290%			
bioPom	+147%	−53%	+5242%	+304%	+679%	+355%	+395%	+724%			
bioVW	−78%	−98%	−99%	−100%	−67%	NF	−100%	−100%			
